# Parents’ perspectives of factors affecting parent–adolescent communication about type 1 diabetes and negotiation of self-management responsibilities

**DOI:** 10.1177/13674935221146009

**Published:** 2022-12-18

**Authors:** Caroline Rawdon, Sophia M Kilcullen, Nuala Murphy, Veronica Swallow, Pamela Gallagher, Veronica Lambert

**Affiliations:** 1 8818 Dublin City University, Dublin, Ireland; 2 11457 Children’s Health Ireland at Temple Street, Dublin, Ireland; 3 111995 Sheffield Hallam University, Sheffield, UK

**Keywords:** adolescent, parents, communication, diabetes mellitus, type 1, self-management

## Abstract

Adolescence is an important time in which young people take on type 1 diabetes (T1D) self-management responsibility. Parents are key facilitators of this process. Little is known about parents’ experiences of communicating with their children about T1D during adolescence. Semi-structured interviews were conducted with 32 parents (24 mothers and 8 fathers) of adolescents (11–17 years) living with T1D to explore how parents communicate about T1D and self-management with their adolescent children. Parents were recruited through two national child and adolescent diabetes and endocrine clinics and online advertisement through a national diabetes advocacy organisation. Interviews were transcribed verbatim and thematically analysed. Six themes were identified: *parent factors, quality of the parent–adolescent relationship, communication strategies, adolescent factors, communication triggers* and *family/system factors*. Understanding factors that impact communication about self-management between parents and adolescents will enable healthcare professionals to provide support and targeted interventions as parent and adolescent roles change over time.

## Introduction

Type 1 diabetes (T1D) is one of the most common chronic conditions in childhood (Mayer-Davis et al., 2018), and requires careful management to maintain good glycaemic control and prevent long-term complications (Cameron et al., 2018). Such management requires commitment and active patient involvement in self-management of lifestyle and acquisition, implementation, and maintenance of complex skills (e.g. frequent self-monitoring of blood glucose levels, self-administration of insulin and balancing insulin requirements with diet and exercise) (Cameron et al., 2018). Self-management of T1D in adolescence comprises three attributes: *process*, *activities* and *goals* (Schilling et al., 2002). Communication between parents and adolescents is part of the activities and process of shared T1D management and transfer of responsibility for T1D management over time (Tuohy et al., 2019; Rawdon et al., 2020).

Sharing of self-management responsibilities between parents and adolescents with T1D is recommended for optimal adolescent and parent outcomes (Cameron et al., 2018). Responsibility-sharing requires daily communication and negotiation, and continuing positive and collaborative parent involvement during transition into adolescence which enhances glycaemic control and quality of life (Jaser, 2011). The importance of effective communication to clarify roles and responsibilities for T1D throughout adolescence was highlighted in a recent scoping review (Gardener et al., 2020). Ideally, as adolescents mature, T1D management moves from parent-dominant through transitional to adolescent-led care, with adolescents assuming increasing responsibility for T1D self-management over time (Schilling et al., 2006; Gan, 2019). Therefore, communication must be flexible enough to negotiate and facilitate gradual handover of increasing amounts of responsibility from parents to adolescents as they mature (Young et al., 2014).

The process of negotiation of T1D responsibilities is complicated in adolescence; a developmental period when adolescents are striving for independence from parents, yet parents need to continue providing support to adolescents to enable adequate monitoring of their health (Monaghan et al., 2015). Indeed, it has been noted that parental monitoring may need to increase rather than decrease during adolescence, depending on adolescent ability (Young et al., 2014). As adolescents become more independent they often feel parents are overprotective (Hung et al., 2020). It is unsurprising therefore that conflict or challenges to parent–adolescent communication can arise (Berg et al., 2017; Campbell et al., 2019), and have a negative effect on adolescent self-management and glycaemic control (Luyckx et al., 2013).

A recent qualitative study exploring family life from all family members– perspectives highlighted a high degree of family involvement in the lives of adolescents with T1D and difficulties with sharing self-management responsibilities (Overgaard et al., 2020). Adolescents highlighted a number of parent-related intrusive behaviours including aspects of parental communication which adolescents perceived as unhelpful, such as nagging, constant questioning and unwanted comments on self-management activities (Overgaard et al., 2020). Findings also indicated difficulties parents face when deciding when to step in or step back to support T1D self-management (Overgaard et al., 2020). While this issue has been previously highlighted and directly impacts parent–adolescent communication about T1D self-management responsibility (Barbler and Strickland, 2015; Haegele et al., 2022), there is still a lack of evidence on parent experiences of communicating about, and negotiating responsibilities for, T1D management with adolescents.

For adolescents living with T1D, characteristics of parent–adolescent communication and parent behaviour can impact adolescent self-management behaviour and feelings about T1D self-management (Holtz et al., 2017; Goethals et al., 2019; Radcliff et al., 2018). Over 10 years ago, Dashiff et al. (2011) interviewed parents of 16–18 year olds about their experiences of supporting adolescents’ T1D self-management. Parents disclosed a number of communication issues which they felt were supportive or not supportive. Examples of supportive communication were reminding and stressing complications, whereas scolding, nagging, checking, judging and getting emotional had a negative impact on adolescent self-management. More recently, Holtz et al. (2021) identified that more information is needed to support parents to balance their involvement in T1D management throughout adolescence and to nurture effective communication throughout this developmental period. While communication between parents and adolescents is recognised as a key feature of transitioning from parent-led to adolescent-led T1D self-management, there is still a lack of understanding around how and why families talk about T1D, conversation contexts, and how communication may support T1D self-management activities and goals and the process of transferring self-management responsibility (Gardener et al., 2020). This study aimed to address this gap to gain a greater understanding of parent perspectives of factors affecting parent–adolescent communication and negotiating T1D management responsibilities during adolescence.

## Aim

To explore parent–adolescent communication and negotiation of self-management responsibility from the perspective of parents of adolescents aged 11–17 years living with T1D.

## Method

This study employed a qualitative descriptive design and semi-structured interviews were conducted. This approach was used to provide a rich description and comprehensive summary of the direct experiences of parents, staying close to data and everyday events (Sandelowski, 2010). This study was conducted and reported in accordance with COREQ – Consolidated Criteria for Reporting Qualitative Research (Tong et al., 2007). See Supplemental File 1. Ethical approval was obtained from Children’s Health Ireland at Temple Street (TSCUH/16.056), Mater Misericordiae University Hospital (MMUH/1/278/1850), and Dublin City University (DCUREC/2016/144) Research Ethics Committees. The study objective was explained to all participants, and written informed voluntary consent was obtained prior to interview.

### Eligibility criteria and recruitment

Inclusion criteria were parents who had an adolescent child aged between 11 and 17 years’ old who was living with T1D for 6 months or longer. Parent for the purpose of this study was defined as a primary caregiver to the adolescent living with T1D, that is, biological parent or legal guardian. Exclusion criteria were parents of adolescents with type 2 or secondary diabetes. Parents were purposively recruited via two national diabetes and endocrine outpatient clinics in the Republic of Ireland – one clinic situated within a children’s hospital and one adolescent clinic situated within an adult hospital. Parents were also recruited via open advertisement on a national diabetes advocacy organisation’s website and social media pages.

### Data collection

All participants completed a demographic information questionnaire. In three cases, both parents from the same family participated with each parent completing their interview separately. The same researcher (author 1), with qualitative research experience, interviewed all participants using a semi-structured interview topic guide developed by the research team’s expert opinion and aligned with study aim. The topic guide was reviewed and refined after completion of two interviews. Parents were asked to describe their experience of learning about their child’s T1D diagnosis, their perception of adolescent’s experience of living with T1D, T1D management roles and responsibilities, and what it was like for them to talk to their adolescent about T1D care and management, including: Why, how, when do you talk about your child’s diabetes? Do you find talking to your child about their diabetes challenging or easy? What do you find challenging or easy to talk about? Can you recall a time when you found it challenging/easy to talk to your child about their diabetes care? What things enable or prevent you from talking about your child’s diabetes? Has the way that you talk about diabetes changed as your child has gotten older? Interviews were audio recorded and ranged in duration from 39–78 min. Interviews either took place in family homes (n = 18), a university room (n = 13) or another location (n = 1), depending on parent choice. On completion of the interview, participants received a debriefing sheet with details of T1D-related supports.

### Data analysis

Interviews were transcribed verbatim, cross-checked for accuracy and de-identified with each parent assigned a number to indicate quotation source. Analysis was carried out in line with Braun and Clarke’s (2006, 2013) six-step thematic analysis framework: data familiarisation, generation of initial codes, searching for themes, reviewing themes, defining and naming themes, and producing the report. NVivo 12 (QSR International Pty Ltd., 2018) was used to assist with data management. Two members of the research team (first and second author) performed data analysis. Both authors were involved in the coding process, each coding 20 and 12 interviews independently. Complete coding was carried out across the interviews and the codes were collated across the entire dataset. Codes were data derived. New codes were generated as required. In line with Braun and Clarke’s (2019) publication, the first and second authors were ‘*collaborative and reflexive*’ (p. 6) in coding the data, cross-checked a sample of coded transcripts from each other, and had frequent and ongoing discussions as data were coded to ensure consistency in coding processes. Following independently coding interview transcripts, two researchers (first and second author) collated all data relating to communication about T1D and T1D management and developed initial themes together through ongoing discussion, via regular meetings, to reach consensus and review, define and name themes jointly as data analysis progressed. Themes were continually checked against the entire data set to ensure they accurately reflected interview content. Themes were reviewed and refined until boundaries between themes were clear.

## Findings

Twenty-four mothers (75%) and eight fathers (25%) of adolescents living with T1D participated (median age 47 years, IQR 9 years). Twenty-three parents were recruited from a clinic in a children’s hospital, three from an adolescent clinic in an adult hospital, and six from open advertisement. Length of time adolescents lived with T1D ranged from one to 12 years (median 6.5, years, IQR 4). All adolescents resided with their parents and therefore findings do not include content regarding transitioning adolescents to self-care away from their parents.

Six themes were identified: *parent factors, quality of the parent*–*adolescent relationship, communication strategies, adolescent factors, communication triggers* and *family/system factors*. The following section presents the themes and should be read together with Table 1 which displays the themes and sub-themes and example quotations to enhance understanding of parent experiences.

**Table 1.** Themes, sub-themes and example quotations.

Themes and sub-themes	Example quotes
Theme 1: Parent factors	
Acceptance of adolescent’s T1D	‘It was really [doctor’s name] whipped us into shape when he took us back in. He just said, ‘you are not dealing with this. You think you are dealing with this, you think you are being terribly clever and managing it but you haven’t accepted it.’ He talked about putting [adolescent’s name] into counselling and we said, ‘are you joking, our child doesn’t need counselling.’ We were very prissy about it; we are not that kind of family. Actually we were exactly that kind of family, we hadn’t accepted it. But once we accepted it and said it was okay, don’t be frightened of it, then we were all right… But until you come to accept that it is not a monster and it is something to be… Well I will say proud, but [adolescent’s name] has to be proud of who he is, every little part of him, the good and the bad’. (parent 29 – mother of a 16-year-old male, diagnosed at age 9)
Understanding adolescent’s lived experience of T1D	‘So we would understand why she has had it and where it has come from and about it and all the rest, we are very in tune with it and with the result she is in tune with it as well. There is nothing that we don’t talk about, we don’t discuss with her because she would ask what happened and how did I get it and I would tell her and I understand that side of it and where her body is going and what is happening to her’. (parent 13 – mother of a 12-year-old female, diagnosed at age 8)
Parental emotional wellbeing	‘Challenging when I am annoyed, and probably for him as well having to listen to me’. (parent 7 – mother of a 15-year-old male, diagnosed at age 11)
	‘Not difficult to communicate because we don’t have any problem communicating at all but very upsetting that she is thinking the things that she is thinking’ (parent 21 – mother of a 16-year-old female, diagnosed at age 5)
T1D impact on parenting	‘…it is parenting plus; it is just an additional bit of parenting. And in many ways, particularly since he has been a teenager, in many ways it has been very helpful as a parent because it has helped us put boundaries around his behaviour and his habits in place’. (parent 29 – mother of a 16-year-old male, diagnosed at age 9)
Theme 2: Quality of the parent–adolescent relationship	
Maintaining close relationships and providing support	‘We have the best relationship a mother and daughter possibly could. I am not gloating in any way saying that, it couldn’t possibly be better so anything [adolescent’s name] ever has on her mind she talks to me about it, no holds barred, no matter what it is. There are open lines of communication the whole time so that is very good from that point of view. We go along our merry way for days and weeks at times and we just get on with it and then she might get very upset about something that has happened, that the whole future is dwelling on how she is going to cope with this and whether she is going to get illnesses to go with it and whatever. So we talk about it and we deal with it and then we move on’. (parent 21 – mother of a 16-year-old female, diagnosed at age 5)
Mutual openness and honesty	‘Yes. I am straight forward with [adolescent’s name]; I don’t hide anything from her. I don’t say oh God I am not telling her that. I am very honest with her and she is honest with me and she will come and ask me things and if I can answer them the right way I do answer them, I don’t answer in a lie to her, I tell her the truth of everything that she wants to know’. (parent 12 – mother of a 13-year-old female, diagnosed at age 12)
	‘So it is hard enough because trying to kind of keep an even keel with teenagers is tough and you want them to be open and you want them to talk to you and keep the lines of communication open but when you have to nag them constantly it turns them off coming to you really you know’. (parent 16 – mother of a 14-year-old female, diagnosed at age 5)
Theme 3: Communication strategies	
Engaging in supportive communication	‘If you are positive with her, if you are positive, if you say if your bloods are good you will feel good. Yeah I think if you are positive with her that is the best way to do it. Like if you are going, “how are you, do you need something?”’ (parent 2 – mother of a 13-year-old female, diagnosed at age 9)
	‘…you have to let them know they can have a normal life like everyone else can have. And I suppose be positive with them and let them appreciate that it is something that they can live with and that they can get on with everything same as everyone else can’. (parent 13 – mother of a 12-year-old female, diagnosed at age 8)
Acknowledging the impact of emotion and timing on communication	‘If we all try and stay calm [laughs] and don’t panic. I think we have learned if something needs to be discussed let’s park it for an hour or two until we all are in the right frame of mind for it’. (parent 15 – mother of a 15-year-old female, diagnosed at age 11)
	‘…I think I have got better because there was one session, again about a year after she was diagnosed and they said the kids don’t want to hear the minute they come in, “what were your bloods?”’ (parent 17 – mother of a 15-year-old female, diagnosed at age 10)
Taking a flexible or adaptable approach to communication	‘Well I suppose this is where it becomes bespoke to the personalities, between my personality and her personality and I am sure no other parent will be exactly the same. I would typically try to leave it go, particularly there is no point in compounding it by letting it become more of an argument because her voice will get raised, I know she won’t listen to me, I will get frustrated and annoyed as well. So certainly more and more as time has gone on I would kind of let it go and come back a bit later’. (parent 26 – mother of a 17-year-old female, diagnosed at age 13)
	‘Well just to try and find a way of communication. If the normal way is not working to try and adapt and find a different way, there is always a different way that you can communicate. And it is a lot to do to know your own child obviously but if the way you would normally do it, if that is not working step back and think about a different way of doing it but don’t give up. Don’t say there is no talking to him. There is a way; you just have to find it’. (parent 19 – mother of a 15-year-old male, diagnosed at age 7)
Actively acknowledging adolescent lived experience	‘So when we talk about diabetes, I mean I suppose she is the tutor and I am the pupil and I learn from her every day…. But I definitely could learn an awful lot from her’. (parent 27 – mother of a 15-year-old female, diagnosed at age 12)
	‘Yes, he said he wanted to just be in control of everything and I said, “yes no problem, because you do actually have the knowledge to do it so you have no reason why you couldn’t do it”’ (parent 19 – mother of a 15-year-old male, diagnosed at age 7)
	‘Breathe, listen to what they are saying. It is very, very easy to get stuck on the, sweet Jesus, I know it all, and they know nothing. No, they are going through it, it is a whole new experience for them, but as a parent and as an adult, you sometimes assume that you know more. You do know more in the grander scale of things because you have more access to information but now that Google is there and they log in and find out things about conditions and it is much more accessible to them’. (parent 22 – mother of a 17-year-old male, diagnosed at age 14)
Refraining from ‘giving out’ (i.e. scold/reprimand) or placing blame	‘So that’s how I… I wouldn’t barrage about him or try not to give out about him but kind of go in a roundabout way of saying, “You kind of need to keep tabs on these things.”’ (parent 11 – mother of a 16-year-old male, diagnosed at age 15)
	‘So you have to kind of pussyfoot around them and just say, “your bloods are high”, and if they go, “well I don’t know why”, say, “I am not blaming you, we just need to figure out why they are high so did you do anything today?”’ (parent 31 – father of a 15-year-old female, diagnosed at age 11)
Theme 4: Adolescent factors	
Adolescents’ understanding of T1D and T1D management	‘He is good to have those conversations; he is very adult about it in his thinking. So he is good, he is okay to talk about it to’. (parent 29 – mother of a 16-year-old male, diagnosed at age 9)
Maturity	‘So as she has gotten older and wanted to know more and asked, we know we can tell her now because she is old enough to understand it. I suppose it is a good time to let her know now that she learns to grow up with it kind of, that kind of knowledge really you know’. (parent 13 – mother of a 12-year-old female, diagnosed at age 8)
Changing adolescent communication patterns resulting from developmental changes	‘Well no you see I would say when she was easier to mind, she was easier to mind when I was completely doing everything because she was so small and she just accepted well I have to take that amount of insulin, mammy is doing it all for me’. (parent 1 – mother of a 13-year-old female, diagnosed at age 3)
	‘I can talk to her it’s just that lately a lot of the time we are talking it is giving out. So she would just say, “I know, I know, I know, I know, I know.” Yeah, she used to be really easy to talk to but not in the last nine months say’. (Parent 2 – mother of a 13-year-old female, diagnosed at age 9)
	‘Probably not. Yeah well when he was diagnosed first because he had a very good then, he was very accurate about everything and didn’t have girlfriends, didn’t have a mobile phone. He was very good then and we talked a lot about diabetes and that is how I think he is kind of comfortable about it, he didn’t worry, off he went out with his Lucozade and didn’t care but now it is a bit tougher. He is getting a bit laid back about it and I just feel I am taking on all the worry now’. (parent 7 – mother of a 15-year-old male, diagnosed at age 11)
	‘Again it is getting harder to offer emotional support because she is at the age where she pulls away from her parents and she has been for the last couple of years. That is the bit I would like to be able to give more of to her but as a parent, a teenager, your parents’ opinions are kind of semi-irrelevant so it is just to always be there to support her if she needs it’. (parent 32 – father of a 15-year-old female, diagnosed at age 8)
	‘But other than that, once there is communication, if there is no communication that is where there is big problems. But as long as you can find a way to communicate these hard years, the teenage years, I think you are on the right track hopefully’. (parent 19 – mother of a 15-year-old male, diagnosed at age 7)
Biological influences	‘What can happen is, as it did here, there a few days ago where his numbers, on Saturday, he woke up and his numbers were way high and we couldn’t explain why and we were like what happened? His numbers were 24 something. And you are trying to figure out what happened but sometimes it can be difficult then because they get a bit harder to talk to if the numbers are high and then you are just trying to get the pump off to see if there is a problem with the pump, load a new pump and he was heading down for a day down… so he was getting ready for that and you are talking to him about it’. (parent 10 – mother of a 14-year-old male, diagnosed at age 6)
	‘Sometimes it can be challenging, especially if he is tired because he will just say, “Oh, I don’t want to talk about this now”’ (parent 19 – mother of a 15-year-old male, diagnosed at age 7)
Adolescents’ willingness to engage in conversations about T1D	‘Challenging would be a good word because as I said [adolescent’s name] doesn’t like to talk about it, [adolescent’s name] doesn’t like having it so she doesn’t really like to discuss it in great depth’. (parent 31 – father of a 15-year-old female, diagnosed at age 11)
	‘She doesn’t express the need for support to us, every so often you will get the roll of the eyes, but yeah she doesn’t come looking for support. Whether she needs it from her friends or not, I don’t get the impression that she does, I don’t get the impression that she talks about it’. (parent 32 – father of a 15-year-old female, diagnosed at age 8)
Theme 5: Communication triggers	
Adolescent questions	‘So yeah she just kind of asks questions, wanting to know… So as she has gotten older and wanted to know more and asked, we know we can tell her now because she is old enough to understand it’. (parent 13 – mother of a 12-year-old female, diagnosed at age 8)
Out-of-range blood glucose readings	‘And she would say to me the odd time, ‘mam I was low after such and such a thing and I took insulin and maybe we need to look at that’. But she would just throw the statement out there, maybe we need to look at it, and then we would follow up on it at some stage but it may not necessarily be there and then, you know’. (parent 24 – mother of a 14 of a year-old female, diagnosed at age 6)
	‘Or if she has been out or out late and the bloods aren’t good, if she hasn’t come home on time, it is kind of an extra thing to nag about because there is enough, with teenagers there is loads [laughs]’. (parent 16 – mother of a 14-year-old female, diagnosed at age 5)
Changes in regimen or routine	‘So as he is getting older and he has it for longer the monitoring and the discussion would get less but at the moment… It’s high at the moment because as I said he’s changed his carbs and he’s changed his routine and the insulin levels are changing hugely.’ (parent 11 – mother of a 16-year-old male, diagnosed at age 15)
Anticipation of upcoming clinic visits	‘I kind of felt we used to, before our visits, we used to write our questions, '[adolescent’s name] what questions do you have?’ Because he would have different questions to what we would have… So we would have a little log of them. And then [adolescent’s name] would have the best question of all, so that would be the one that is obviously asked when we get in there. And then each time we would leave with a bit of relief’. (parent 3 – mother of a 14-year-old male, diagnosed at age 11)
	‘The only time we have a big, oh we have to get on top of the diabetes, is generally when we know there is a [hospital name] appointment in about three weeks away and we are like, we are in trouble here, I would say your blood sugars are quite high, we need to re-focus here’. (parent 25 – mother of a 15-year-old female, diagnosed at age 11)
Adolescents getting upset or emotionally overwhelmed	‘She might get very upset about something that has happened, that the whole future is dwelling on how she is going to cope with this and whether she is going to get illnesses to go with it and whatever. So we talk about it and we deal with it and then we move on’. (parent 21 – mother of a 16-year-old female, diagnosed at age 5)
Theme 6: Family/system factors	
Family routines	‘Probably the fact that we sit together for breakfast and for dinners and he’s monitoring himself alongside of us. It’s very open in the house. He doesn’t go away to his room to check his bloods and to take insulin and stuff like that so it’s very open, his diabetes and his treatment’. (parent 11 – mother of a 16-year-old male, diagnosed at age 15)
Attendance at clinic appointments	‘I suppose the information I got in the hospital would give me a certain knowledge about it. And that is probably it really, trying to talk sense into her and giving my point of view and that if she doesn’t believe me or if she doesn’t think what I am saying is right that we will ask the next time we are in the hospital and generally she would take on board whatever I would say to her’. (parent 30 – father of a 13-year-old female, diagnosed at age 12)
Time	‘Time, basically, I do shift work so I do ten hour shifts, and that is not making an excuse but I might be gone in the morning before they get up for school and when I come in the evening then she might be gone out with her friends and then she might come in maybe before she goes to bed…’ (parent 30 – father of a 13-year-old female, diagnosed at age 12)

### Parent factors

Parents identified a number of personal factors relating to their own thoughts, feelings and behaviour that influenced how they communicated with adolescents about T1D. Parental *acceptance of adolescent’s T1D* was an important precursor to meaningful communication about T1D. Acceptance by parents facilitated normalisation of T1D and engendered an understanding that T1D is a part of but does not define adolescents themselves. An enabler of parent–adolescent communication was parents’ *understanding adolescents lived experience of T1D*, such as T1D bodily effects and self-management activities and associated equipment requirements*. Parental emotional wellbeing* could impact the process of communicating about T1D. A high value was placed on communication and parents saw it as an essential support for adolescent wellbeing. Some parents viewed T1D as an extension to normal parenting responsibilities rather than a major burden, thereby maintaining a positive outlook on *T1D impact on parenting*. Other parents described how T1D facilitated communication with adolescents about rules and expectations around adolescent behaviour.

### Quality of the parent–adolescent relationship

Some parents spoke of *maintaining close relationships and providing support* to ensure that adolescents always knew that they were there for them if they wanted to talk, or needed affection and reassurance. Other parents highlighted *mutual openness and honesty* as important, when talking about T1D. Openness to discussing negative feelings and difficult topics, as well as positive ones, was highlighted by parents and this extended to communicating about potential future negative consequences of T1D and how these could be avoided, and included parents being able to express their own worries to adolescents. Maintaining a good quality relationship involved a delicate balance of keeping lines of communication open without causing conflict or alienating adolescents by coming across as ‘nagging’. One mother described how she and her daughter with T1D vented frustrations but she felt able to apologise when communication did not go well. Even if conflict ensued, a close relationship was able to withstand such encounters if openness was maintained, such that both parties were quick to move past their differences.

### Communication strategies

Parents described a number of strategies they employed when communicating about T1D or planning to communicate about T1D with their adolescent to ensure smooth communication and to encourage adolescent self-management efforts. *Engaging in supportive communication* (e.g. taking a positive outlook, encouraging, praising and active problem-solving) included communicating positively about self-management benefits and living well with T1D if adolescents learnt to control their blood sugars. Some parents described taking a positive outlook on T1D as important when communicating with adolescents to let them know that T1D was something that they could live with and it would not restrict their life. Other parents were positive in their communication by acknowledging and celebrating adolescents performing well in their self-management. One mother explained how she did this by communicating enthusiastically and in an encouraging way with her son. Some parents described how they engaged in active problem-solving with adolescents when communicating about T1D management, indicating collaborative parent–adolescent communication. An example is when parents ask questions to help adolescents learn T1D self-management skills such as figuring out insulin doses themselves.

*Acknowledging the impact of emotions and timing on communication* parents recognised that an aspect of facilitating positive interactions with adolescents was managing emotions, for example, remaining calm or communicating in a calm manner. Being calm facilitated better parent–adolescent communication and some parents spoke about how they preferred to delay communication about T1D to a time when both parent and adolescent were in a more receptive state. Some parents reflected on specific times when they avoided asking about T1D in an attempt to not make every conversation about T1D management, for example, when their child arrives home from school. Parents employed strategies such as asking about something else before asking adolescents about blood glucose control throughout the day.

*Taking a flexible or adaptable approach to communication* with their adolescent revealed itself as an important strategy in facilitating communication between parents and adolescents. Reflecting on how interactions had gone and adapting to improve parent–adolescent communication was also highlighted as important by some parents. Parents used their judgement of situations and their knowledge of their adolescent (personality and circumstances) to decide what approach to take to communication. Some parents advised that they reflected on whether their communication approach was working and if not they changed their style.

*Actively acknowledging adolescent lived experience* when talking about T1D was identified by parents as having a facilitative effect on parent–adolescent interactions. Although it was easy for parents to assume they potentially knew more than the adolescent, young people had easy Internet access to T1D-related information. Acknowledging adolescents T1D knowledge as, or before, a parent put forward their own views, facilitated smoother communication, and meant parents listened to adolescents. Parents recounted recognising and confirming adolescent competence in self-management when communicating about adolescent’s requested handover of responsibility.

Other parents described taking a more indirect approach to engaging with adolescents about T1D and self-management which *refrained from ‘giving out’ (i.e. scold/reprimand) or placing blame*. Parents described how they supported adolescents by reminding them of self-management responsibilities without *‘giving out’*, for example, one father described how he supported his daughter by engaging in conversation about high blood glucose and why this was occurring without blaming her. Other parents described how they employed strategies to communicate important information to adolescents, such as providing prompts for carbohydrate counts in school lunch boxes and letting adolescents read their hospital correspondence or other T1D-related information for the first time. These practical strategies were more commonly used when adolescents were younger.

### Adolescent factors

A number of adolescent factors which impact on parent–adolescent communication were identified by parents. *Adolescent’s understanding of T1D and T1D management* and its range of implications were often pivotal to effective parent–adolescent communication. Some parents connected this understanding with adolescent age and *maturity* level and their readiness to receive and understand information. This maturity was described by parents as adolescents being ‘adult’ about T1D, accepting T1D and being easy to talk to. Parents explained how as their adolescent got older they perceived them as growing more mature and capable of understanding T1D and its implications, and they also judged them as more ready to receive information – both of which facilitated communication about T1D.

*Changing adolescent communication patterns resulting from developmental changes,* such as an increased strive for independence, often facilitated communication about T1D. However, in contrast to parents who stated that communication got easier as children got older, some parents believed talking about T1D issues with their child was easier when children were younger because they accepted parental directions more readily and were okay with parents assuming full responsibility. Some parents recounted how adolescents increasingly challenged what they said as they got older. Over time, adolescents were less interested in hearing what parents had to say and interactions were described as *‘giving out’*. Consequently, some parents described a decrease in conversation frequency as adolescents got older. The comparative ease of communication when children were younger was facilitated by lack of distractions in children’s lives. Parents described how normal age-related issues came into play and acted as barriers to discussion of T1D. Parents perceived a shift in attention or care adolescents paid to looking after their T1D and that the majority of burden of concern lay with parents. Many parents described difficulties when trying to communicate T1D importance during the teenage years. Some parents connected difficulty getting adolescents to engage in conversations related to T1D issues with typical challenges involved in communicating with teenagers. Parents pointed out that maintaining communication with adolescents, even if limited or difficult, was better than no communication at all. In some instances, adolescents requested more independence in their T1D self-management.

*Biological influences,* such as out-of-range blood glucose and fatigue, also impacted on parent–adolescent communication. Parents explained how sometimes adolescents were more difficult to talk to when their blood glucose was high. Other parents pointed out that tiredness or fatigue also made communication difficult with adolescents less likely to engage in conversation when feeling tired. *Adolescent’s willingness to engage in conversations about T1D* was also identified by parents as a barrier to communication at times, with some parents describing adolescents’ reluctance to engage in conversations about T1D, which may be affected by emotional issues including coping, adjustment and self-esteem.

### Communication triggers

A number of issues or scenarios acted as triggers for parent–adolescent communication about T1D. *Adolescent questions* about T1D and its management instigated conversations. *Out-of-range blood glucose readings* were a trigger for parents communicating with adolescents. Sometimes communication about out-of-range blood glucose readings could evolve into *‘nagging’* adolescents rather than a discussion with them. *Changes in regimen or routine*, such as changes in carbohydrate intake and insulin regimen could also trigger an increased frequency in discussions between adolescents and parents for particular time periods. *Anticipation of upcoming clinic visits* also triggered parent–adolescent communication in advance of meeting HCPs. A number of parents described instances of *adolescents getting upset or emotionally overwhelmed* and how this led to conversations. Some parents spoke about engaging in conversation when their adolescent expressed worry about T1D.

### Family/system factors

Parents talked about a number of wider family-related or systemic factors that could facilitate or constrain communication with adolescents about T1D. *Family routines* such as sharing meals together often facilitated communication between parents and adolescents. Knowledge about T1D derived by both parents and adolescents from diabetes team interactions through *attendance at clinic appointments* together facilitated communication between parents and adolescents. By contrast, a lack of *time* due to external work demands and competing needs of other children in the family could lessen opportunities for, or constrain communication about T1D.

## Discussion

This study explored parents’ perspectives on communication about T1D and T1D self-management with their adolescent children. Six themes were identified: *parent factors, quality of the parent*–*adolescent relationship, communication strategies, adolescent factors, communication triggers* and *family/system factors*. Findings provide important information for supporting parents to nurture effective communication throughout adolescence, including, understanding how timing and one’s own emotions may impact communication; nurturing a positive relationship with adolescents; and employing strategies to maintain positive communication. This reflects other evidence which highlights a positive impact on adolescent diabetes behaviour and life quality through fostering positive communication with parents (Holtz et al., 2020), and building a trusting parental relationship through communications to decrease conflict (Babler and Strickland, 2015).

We found that awareness of contextual cues and mechanisms of parent–adolescent communication in this population can help increase understanding of parent–adolescent communication dynamics and awareness of potential communication process intervention points, for instance, helping parents to understand developmental changes which may impact communication during adolescence; supporting parents as they manage their own feelings about adolescent T1D diagnosis; and guiding parents to use strategies which may be effective in managing communication.

To our knowledge, this is the first study to exclusively investigate communication-based strategies that parents use when discussing T1D and T1D self-management with adolescents. Findings indicate that parent–adolescent communication from parents’ perspectives reflects a multi-dimensional and reciprocal process that includes factors attributable to parents and adolescents as well as wider environmental issues.

This study highlights that parents employed a number of strategies when communicating about T1D management. One strategy included engaging in supportive communication (e.g. taking a positive outlook, encouraging, praising and active problem-solving with adolescents). Timing communications appropriately, managing emotions, and being flexible and adaptable were important parental considerations for facilitating positive T1D interactions. These findings help to address a relative dearth in literature by advancing understanding of more adaptive communication strategies used by parents when interacting with adolescents. Parents actively acknowledged adolescent lived experience when approaching issues relating to T1D management. This supports Goethals et al.’s (2019) study reporting an association with better T1D self-management when parents explain personal relevance of self-management advice, accept adolescent perspectives, and facilitate adolescent choice and initiative in their interactions.

Although some parents acknowledged the tendency to *‘nag’* at times, many parents reported refraining from *‘giving out’* and avoiding placing blame when self-management was not optimal. In a study by Holtz et al. (2017), however, no parents talked about using positive strategies to communicate with children about T1D self-management. While parents had good intentions to communicate positively with children about T1D, any conscious effort in communicating positively was hindered by parents’ need to know their child’s blood glucose levels. Similar to Dashiff et al.’s (2011) findings, parents in our study reported that using a strategy of reminding adolescents of self-management responsibilities can facilitate better self-management but can also be perceived by adolescents as negative and controlling. Parental negative and controlling behaviours are associated with poorer T1D-related and psychosocial outcomes (Gruhn et al., 2016). A further strategy reported by Dashiff et al. (2011), in support of our study, was that parents expressed an appreciation and understanding of the adolescent’s T1D self-management through praise and positive reinforcement; strategies which have been previously linked to better psychological adjustment and glycaemic control (Jaser and Grey, 2010).

To date much research in this area has focused on how communication concepts such as warmth, control, support and conflict impact on adolescent wellbeing and T1D outcomes (Dashiff et al., 2008), importance of communication tone used by parents and communication frequency (DeBoer et al., 2017), and autonomy-supportive versus controlling styles of interaction (Goethals et al., 2019). Our study adds to this knowledge base by enhancing understanding of parent–adolescent communication triggers regarding T1D and self-management. Triggers which facilitated communication included upcoming attendance at T1D clinic appointments, out-of-range blood glucose levels and changes in regimen. Additionally, ongoing adjustment to T1D from adolescent perspectives was reported to have an impact on communication and specific emotional events or negative feelings could trigger adolescents to talk about T1D. Knowledge about triggers of parent–adolescent communication can help parents better prepare for interactions with adolescents and optimise opportunities for improving parent–adolescent trust and disclosure, relationship quality, adolescent self-efficacy and self-management – factors associated with better T1D outcomes in adolescents (Osborn et al., 2013; Tsiouli et al., 2013).

Parents noted that adolescent development and maturity also impacted communication about T1D management. There is a need for more nuanced information about changes in family communication and relationship patterns during adolescence for adolescents living with T1D. Developmentally normative changes occur in parent–adolescent relationships throughout adolescence (Pihlaskari et al., 2020; Williams et al., 2014) that can influence parent–adolescent communication, adolescent self-management and glycaemic control (Almeida et al., 2020). Parents in this study indicated that they were more likely to initiate communication with adolescents if they perceived them to be developmentally ready; when adolescents were older parents were more prepared to talk openly about possible negative consequences of T1D. One feature of adolescents’ growing maturity reported by some parents was a decrease in conversation frequency as adolescents got older due to increasing distractions in a teenager’s life. This finding resonates with research in broader developmental literature indicating that adolescent disclosure decreases across early adolescence in particular, and that adolescents actively push the balance towards less frequent communication to meet their growing privacy and autonomy needs (Keijsers and Poulin, 2013). However, a sequential cohort study of parents and adolescents with T1D examining decision-making involvement in T1D self-management found that the extent to which adolescents seek advice regarding T1D, express opinions and give information tends to increase as they mature (Miller and Jawad, 2019).

Some parents in our study reported increasing adolescent resistance to advice or rules. Goethals et al. (2019) indicated that a controlling parenting style leads to adolescent defiance against T1D self-management. However, parents in our study also reported figuring out ways to negotiate with adolescents to arrive at a compromise in key situations regarding self-management, in line with a more autonomy-supportive and collaborative approach to communication (Goethals et al., 2019) – for example, telling adolescents they would allow them to stay overnight with a friend or relative as soon as adolescents learnt how to administer insulin independently. We cannot determine with any certainty whether these differences between findings across studies are culturally related.

Our findings show that conversations about self-management responsibilities are prompted by adolescents as they mature and they tell parents they want to exercise more independence in their self-management regimen. Information about such changes in communication and relationship patterns in adolescence can be helpful for parents who are having difficulty distinguishing what is typical behaviour for an adolescent living with T1D. Parents in this study also identified aspects of their relationship with adolescents which could facilitate or hinder effective communication about T1D and T1D management regardless of adolescent maturity or developmental stage. This included relationship qualities such as closeness, support, openness and honesty. Indeed, relationship quality as a key construct in parental involvement in adolescent diabetes management has been noted as important (Palmer et al., 2011; Young et al., 2014).

Parents also highlighted family or system factors which played a role in communication about T1D; these were aspects of family life that could facilitate communication about T1D such as sharing meals together, or parents having sufficient time to discuss adolescent’s T1D management with them. Time spent together in family activities has predicted higher rates of adolescent disclosure to parents (Willoughby and Hamza, 2011). Disclosure helps parents to stay involved in adolescent’s self-management and provide support when needed (Berg et al., 2017). It is advisable for clinicians to work with parents to help them identify spaces within busy family schedules for talking with adolescents about T1D self-management as well as about more general matters. Our findings illustrate parent–adolescent communication as reciprocal in nature, youth disclosure of information as important and how adolescent willingness to engage with parents is a determinant of the communication process.

### Limitations

While parents were recruited from national adolescent diabetes and endocrine clinics and a national diabetes advocacy agency, a limitation is that self-selection bias cannot be out-ruled because the sample is limited to parents who willingly volunteered to take part. Also, fathers are under-represented; however, it is interesting to note that where fathers (n = 8) did participate, they typically were fathers of boys (n = 5). In instances where fathers of girls took part (n = 3), mothers also took part. There was also limited diversity in the sample in relation to family structures which cautions representativeness of our sample. Further research is needed to target more diversity and complexity in adolescents’ families to take account of changes over past decades in family forms and their prevalence (Pearce et al., 2018). We also acknowledge that further exploration of cultural differences in parent–adolescent communication about T1D management across international contexts, as well as families from different cultural backgrounds in Ireland, is warranted given that the participant profile in this study were all white Irish families. Additionally, in our sample there was wide variability in the length of time adolescents lived with T1D (from one to 12 years) and further quantitative research would be useful to investigate whether parents’ years of diagnosis experience and T1D management affects their communication. This study was also limited in understanding parent–adolescent communication about factors known to impact blood glucose levels such as weight management and alcohol consumption. These factors were not a strong feature in this study’s data and may be a useful line of inquiry to explore in future work. Finally, our findings are drawn from parental reports and do not include adolescent experiences which may differ. However, this study is part of a wider research programme where work is ongoing in exploring adolescent perspectives.

### Implications for practice

The study highlights the importance of appropriate multi-disciplinary services for parents, such as access to a psychologist or clinical nurse specialist, to support and guide parent–adolescent communication and negotiation of self-management responsibility throughout adolescence as roles and responsibilities change. The findings will be helpful for other parents for whom communication with adolescents living with T1D is challenging. Clinicians can discuss and model supportive communication strategies used by parents of adolescents with T1D as potential methods for maintaining warm relationships, building adolescent self-esteem and competency regarding self-management, and managing conflicts when talking about T1D. Moreover, since nagging and other negative communication practices are often underpinned by parental stress and emotions like anxiety (Sweenie et al., 2014), clinicians should consider resources to support parents in managing anxiety and stress, such as mindfulness practice (e.g. Aalders et al., 2018; Burgdorf et al., 2019), and facilitation of parent peer support (e.g. Sullivan-Bolyai et al., 2016). Findings are also important to inform future design and provision of family-focused care interventions to support effective parent–adolescent communication.

## Conclusion

This study contributes unique knowledge into ways parents experience communication with adolescents (11–17 years) about T1D and T1D self-management. Findings highlight areas which HCPs could focus on to support parents to foster effective parent–adolescent communication. Future efforts to improve parent–adolescent communication will benefit from consideration of its multi-dimensional nature, by targeting ways to increase overall parent–adolescent relationship quality, as well as fostering greater awareness of triggers for parent–adolescent communication, and parent-related and adolescent-related characteristics that influence the communication process. Findings will benefit parents with strategies to support effective communication with adolescents living with T1D.

## Supplemental Material

Supplemental Material - Parents’ perspectives of factors affecting parent–adolescent communication about type 1 diabetes and negotiation of self-management responsibilities Supplemental Material for Parents’ perspectives of factors affecting parent–adolescent communication about type 1 diabetes and negotiation of self-management responsibilities by Caroline Rawdon, Sophia Kilcullen, Nuala Murphy, Veronica Swallow, Pamela Gallagher, Veronica Lambert in Journal of Child Health Care
