# Supplementary material for: Parents’ perspectives of factors affecting parent–adolescent communication about type 1 diabetes and negotiation of self-management responsibilities
Source: J Child Health Care. 2022 Dec 18;28(3):514–35. doi: 10.1177/13674935221146009 (PMC11459864; doi:10.1177/13674935221146009)
Supplement: Supplemental Material - Parents’ perspectives of factors affecting parent–adolescent communication about type 1 diabetes and negotiation of self-management responsibilities [file sj-pdf-1-chc-10.1177_13674935221146009.pdf]

Supplementary file 1

Consolidated criteria for reporting qualitative studies (COREQ): 32-item checklist

**Domain and Items**	**Guide questions/description**	**Page Reported on/Author Comment**
**Domain 1: Research team and reflexivity**		
Personal characteristics		
1. Interviewer/facilitator	Which author/s conducted the interview or focus group?	Page 6 (data collection)<br>Author 1 conducted the interviews
2. Credentials	What were the researcher's credentials? *E.g. PhD, MD*	Not reported in the manuscript: five authors/researchers credentials were PhD, one author was MD
3. Occupation	What was their occupation at the time of the study?	Not reported in the manuscript: three authors/researchers were from psychology, two from nursing, and one medical consultant
4. Gender	Was the researcher male or female?	Not reported in the manuscript for gender neutral language: all authors/researchers were female.
5. Experience and training	What experience or training did the researchers have?	Page 6 (data collection)<br>The researcher had experience in qualitative research including conducting semi-structured interviews.
Relationship with participants		
6. Relationship established	Was a relationship established prior to study commencement?	Not reported in manuscript: no relationship was established with participants prior to study commencement.
7. Participant knowledge of the interviewer	What did the participants know about the researcher? e.g. *personal goals, reasons for doing the research ?*	Page 5 (method)<br>The study objective was explained to all participants, this included details of the research team.
8. Interviewer characteristics	What characteristics were reported about the interviewer/facilitator? e.g. *Bias, assumptions, reasons and interests in the research topic*	Not reported in manuscript: the participants were provided with an explanatory statement in the study plain language statement about the research aims and objectives and details on the interviewer and research team.
**Domain 2: study design**		
Theoretical framework		
9. Methodological orientation and theory	What methodological orientation was stated to underpin the study? e.g. *grounded theory, discourse analysis, ethnography, phenomenology, content analysis*	Page 5 (method)<br>A qualitative descriptive approach underpinned the study in order to provide a straight description and comprehensive summary of the direct experiences of parents, staying close to the data and everyday events.

Participant selection		
10. Sampling	How were participants selected? e.g. *purposive, convenience, consecutive, snowball*	Page 6 (recruitment)<br>Participants were purposively recruited
11. Method of approach	How were participants approached? e.g. *face-to-face, telephone, mail, email*	Page 6 (recruitment)<br>Participants were approached in-person in outpatient clinics and through open online advertisement
12. Sample size	How many participants were in the study?	Page 8 (findings)<br>There were 32 participants in the study, 24 mothers and 8 fathers.
13. Non-participation	How many people refused to participate or dropped out? Reasons?	Not reported in the manuscript: Approximately 27 participants did not wish to participate. Some reasons for non-participation were family competing demands, illness, and State examinations.
Setting		
14. Setting of data collection	Where was the data collected? e.g. *home, clinic, workplace*	Page 7 (data collection)<br>The interviews were either conducted in the family home, a room in the university or another location depending on parent choice.
15. Presence of non-participants	Was anyone else present besides the participants and researchers?	Not reported in manuscript: no one else was present besides the participant and researcher.
16. Description of sample	What are the important characteristics of the sample? e.g. *demographic data, date*	Characteristics of the sample reported on page 8 [results]
Data collection		
17. Interview guide	Were questions, prompts, guides provided by the authors? Was it pilot tested?	Page 6/7 (data collection) provides an overview of interview questions which were developed by the research team's expert opinion aligned with the gaps in literature and study aims and objectives. The interview questions and prompts were reviewed and refined after the first two interviews were completed.
18. Repeat interviews	Were repeat interviews carried out? If yes, how many?	Not reported in the manuscript: the interview was once only for each participant.
19. Audio-visual recording	Did the research use audio or visual recording to collect the data?	Page 7 (data collection)<br>The interviews were audio-recorded to collect the data
20. Field notes	Were field notes made during and/or after the interview or focus group?	Not reported in manuscript. Field notes were recorded after each interview noting contextual details

		related to space, mood, any signs of upset etc.
21. Duration	What was the duration of the interviews or focus group?	Page 7 (data collection)<br>Approximate duration of the interviews were between 39 and 78 minutes.
22. Data saturation	Was data saturation discussed?	Data saturation was not discussed as it does not align with the values and assumptions of the reflective thematic analysis approach reported in manuscript (Braun and Clarke 2019)
23. Transcripts returned	Were transcripts returned to participants for comment and/or correction?	Not reported in the manuscript: transcripts were not returned to participants for comment and/or correction
**Domain 3: analysis and findings**		
Data Analysis		
24. Number of data coders	How many data coders coded the data?	Page 7 (data analysis)<br>Two (CR, SK) coders were involved in the data coding process
25. Description of the coding tree	Did authors provide a description of the coding tree?	The coding tree was not reported in the manuscript.
26. Derivation of themes	Were themes identified in advance or derived from the data?	Page 7 (data analysis)<br>Codes and themes were data derived
27. Software	What software, if applicable, was used to manage the data?	Page 7 (data analysis)<br>NVivo 12 (QSR International) software was used to manage data
28. Participant checking	Did participants provide feedback on the findings?	Not reported in manuscript: participants did not provide feedback on findings
Reporting		
29. Quotations presented	Were participant quotations presented to illustrate the themes/findings? Was each quotation identified? e.g. *participant number*	Selected participant quotations were presented in Table 1 to illustrate each theme/sub-theme. Each quotation was identified with a participant/parent number.
30. Data and findings consistent	Was there consistency between the data presented and the findings?	Pages 8-21 (findings and discussion)<br>Yes there was consistency between data presented and findings
31. Clarity of major themes	Were major themes clearly presented in the findings?	Six major themes were presented in the findings on pages 9-14
32. Clarity of minor themes	Is there a description of diverse cases or discussion of minor themes?	Yes there is description of minor sub-themes within the six major themes on pages 9-14

Developed from: Tong, A, Sainsbury, P, Craig, J. (2007) Consolidated criteria for reporting qualitative research (COREQ): a 32-item checklist for interviews and focus groups. International Journal for Quality in Health Care 19(6): 349-357.
